# RETRACTED: Asfour et al. Amitriptyline-Based Biodegradable PEG-PLGA Self-Assembled Nanoparticles Accelerate Cutaneous Wound Healing in Diabetic Rats. *Pharmaceutics* 2022, *14*, 1792

**DOI:** 10.3390/pharmaceutics16010152

**Published:** 2024-01-22

**Authors:** Hani Z. Asfour, Nabil A. Alhakamy, Osama A. A. Ahmed, Usama A. Fahmy, Mohamed A. El-moselhy, Waleed Y. Rizg, Adel F. Alghaith, Basma G. Eid, Ashraf B. Abdel-Naim

**Affiliations:** 1 Department of Medical Microbiology and Parasitology, Faculty of Medicine, King Abdulaziz University, Jeddah 21589, Saudi Arabia; hasfour@kau.edu.sa; 2 Department of Pharmaceutics, Faculty of Pharmacy, King Abdulaziz University, Jeddah 21589, Saudi Arabia; nalhakamy@kau.edu.sa (N.A.A.); oaahmed@kau.edu.sa (O.A.A.A.); uahmedkauedu.sa@kau.edu.sa (U.A.F.); wrizq@kau.edu.sa (W.Y.R.); 3 Center of Excellence for Drug Research and Pharmaceutical Industries, King Abdulaziz University, Jeddah 21589, Saudi Arabia; 4 Mohamed Saeed Tamer for Pharmaceutical Industries, King Abdulaziz University, Jeddah 21589, Saudi Arabia; 5 Department of Clinical Pharmacy and Pharmacology, Ibn Sina National College for Medical Studies, Jeddah 22413, Saudi Arabia; m_moselhy64@yahoo.com; 6 Department of Pharmacology and Toxicology, Faculty of Pharmacy, Minia University, Minia 61519, Egypt; 7 Department of Pharmaceutics, College of Pharmacy, King Saud University, Riyadh 11451, Saudi Arabia; afalghaith@ksu.edu.sa; 8 Department of Pharmacology and Toxicology, Faculty of Pharmacy, King Abdulaziz University, Jeddah 21589, Saudi Arabia; beid@kau.edu.sa

*Pharmaceutics* retracted the article “Amitriptyline-Based Biodegradable PEG-PLGA Self-Assembled Nanoparticles Accelerate Cutaneous Wound Healing in Diabetic Rats” [1] cited above. Following publication, concerns were brought to the attention of the publisher regarding the duplication of images.

Adhering to our complaints procedure, an investigation was conducted, which confirmed the duplication of Figure 1A in [1] with parts of Figure 5A in [2], Figure 2A in [3], and Figure 3A in [4], presenting different experimental conditions. Furthermore, the sub-figures overlapped in Figure 3 in [1], although they had different coloration.

While the authors fully cooperated with the Editorial Office during the investigation, they were unable to satisfactorily explain the overlap of the figures and meet the required quality standards for raw images in order to consider a correction as per the journal’s original image requirements policy (https://www.mdpi.com/journal/pharmaceutics/instruc-tions#oriimages). As a result, the Editorial Board and Editor-in-Chief were unable to confirm the reliability of the findings and subsequently decided to retract this paper.

This retraction was approved by the Editor-in-Chief of the journal *Pharmaceutics*.

The authors did not agree to this retraction. 
